# Temperature Characteristics of a Pressure Sensor Based on BN/Graphene/BN Heterostructure

**DOI:** 10.3390/s19102223

**Published:** 2019-05-14

**Authors:** Mengwei Li, Teng Zhang, Pengcheng Wang, Minghao Li, Junqiang Wang, Zewen Liu

**Affiliations:** 1 Key Laboratory of Instrument Science & Dynamic Measurement, North University of China, Taiyuan 030051, China; sxzhangteng@163.com (T.Z.); wpcyond@163.com (P.W.); limhup@163.com (M.L.); 2 North University of China, Academy for Advanced Interdisciplinary Research, Taiyuan 030051, China; 3 Institute of Microelectronics, Tsinghua University, Beijing 100084, China; liuzw@tsinghua.edu.cn; 4 Microsystem Integration Center, North University of China, Taiyuan 030051, China; wangjunqiang210@163.com

**Keywords:** graphene, heterojunction, temperature characteristics, electrophonon coupling

## Abstract

Temperature is a significant factor in the application of graphene-based pressure sensors. The influence of temperature on graphene pressure sensors is twofold: an increase in temperature causes the substrates of graphene pressure sensors to thermally expand, and thus, the graphene membrane is stretched, leading to an increase in the device resistance; an increase in temperature also causes a change in the graphene electrophonon coupling, resulting in a decrease in device resistance. To investigate which effect dominates the influence of temperature on the pressure sensor based on the graphene–boron nitride (BN) heterostructure proposed in our previous work, the temperature characteristics of two BN/graphene/BN heterostructures with and without a microcavity beneath them were analyzed in the temperature range 30–150 °C. Experimental results showed that the resistance of the BN/graphene/BN heterostructure with a microcavity increased with the increase in temperature, and the temperature coefficient was up to 0.25%°C^−1^, indicating the considerable influence of thermal expansion in such devices. In contrast, with an increase in temperature, the resistance of the BN/graphene/BN heterostructure without a microcavity decreased with a temperature coefficient of −0.16%°C^−1^. The linearity of the resistance change rate (Δ*R*/*R*)–temperature curve of the BN/graphene/BN heterostructure without a microcavity was better than that of the BN/graphene/BN heterostructure with a microcavity. These results indicate that the influence of temperature on the pressure sensors based on BN/graphene/BN heterostructures should be considered, especially for devices with pressure microcavities. BN/graphene/BN heterostructures without microcavities can be used as high-performance temperature sensors.

## 1. Introduction

Graphene and its composites have become an important frontier of international research owing to their excellent electrical, mechanical, and thermal properties, and their two-dimensional structures [1,2,3,4,5]. Among nanoscale carbon materials, monolayer graphene, multilayer graphene, and carbon nanotubes are widely used. Multilayer graphene is obtained by stacking Bemal (AB) in the z direction by a single layer of graphene. The layer spacing of multilayer graphene is 0.335 nm, and it is more isotropic than monolayer graphene. Carbon nanotubes can be regarded as closed nanotube bodies formed by bending single or multi-layer six-carbon ring graphite layers. According to the number of graphene layers, carbon nanotubes are divided into single-walled carbon nanotubes (SWNTs) and multi-walled carbon nanotubes (MWNTs). SWNTs have a good electrical conductivity, with a bundled resistivity as low as 0.06 mΩ·cm [6], and the resistivity of a single MWNT at room temperature is 0.08 Ω·cm [7]. Numerous studies have been conducted on monolayer graphene. Pereira V M et al. analyzed the effect of tensional strain on the electronic structure of graphene. In the absence of electron–electron interactions, within linear elasticity theory, and a tight-binding approach, they observed that strain can generate a bulk spectral gap [8]. Sanaeepour M et al. proposed a nanometer-sized graphene pressure sensor. Mechanical behavior is simulated by means of geometric nonlinear analysis and clamped boundary conditions along all sides of the graphene membrane [9]. Single-layer graphene has excellent electrochemical and mechanical properties, but defect-free single-layer graphene has a low manufacturing rate. Compared with single-layer graphene, nanocomposites of graphene have the advantages of low cost and mass production. Pietro C et al. researched graphene nanoplatelets, hybrids between graphene and graphite [10]. Such nanomaterials have the advantages of planar structure, good thermal conductivity, low cost, etc., and can be applied in the fields of flexible electronics, strain sensors and capacitive sensors [11]. Tian H et al. studied a flexible, wide-range, ultra-sensitive foam structure resistive pressure sensor based on laser-engraved graphene (LSG). The sensitivity of the pressure sensor is as high as 0.96 kPa^-1^ over a wide pressure range (0–50 kPa) [12]. Tao LQ et al. proposed a paper-based high-performance pressure sensor to further improve the sensitivity of the pressure sensor. The sensitivity of the pressure sensor is as high as 17.2 kPa^−1^ over a wide pressure range (0–20 kPa) [13]. Owing to the disadvantages of traditional silicon carbide (SiC) and silicon on sapphire (SOS) PN junction failures at high temperatures, we have proposed a graphene–boron nitride (BN) heterojunction pressure sensor based on the piezoresistive effect, which indicates that BN layers are good passivation layers for graphene devices [14]. Boron nitride hexagonal is a structural analog of graphene with a broadband gap of approximately 5.97 eV [15,16]. BN is a typical anisotropic material with good insulating, thermal and chemical stability. It has high thermal conductivity [60 W/(m·K)], low thermal expansion coefficient [2.6 × 10^−4^] and high tensile strength (41 Mpa) in the direction perpendicular to the c-axis. It has a lower thermal conductivity [1.9 W/(m·K)] and a higher compressive strength in the direction parallel to the c-axis [17,18]. As the difference between the lattice constants of graphene and boron nitride is only 1.7%, theoretical calculation predicts that they can form an in-plane heterostructure [19]. In addition, the heterogeneous interface will exhibit special electronic properties, such as band gap opening, magnetism, excellent heat conduction, and interface electron reconstruction. A novel sensor based on BN and a graphene lattice is proposed. The heterojunction formed by the upper and lower BN layers protects the graphene to reduce carrier heterogeneity and intrinsic doping, thus improving the electrical performance and sensitivity of the sensor.

In practical applications, graphene and its composites are easily subjected to thermal stress and thermal deformation owing to large changes in their ambient temperature; hence, temperature is often a non-negligible factor for graphene pressure sensors, and has a considerable impact on BN/graphene/BN heterojunctions. There are few studies on the temperature characteristics of a sensor based on BN/graphene/BN heterojunction. This study aims to analyze this problem. For the temperature characteristics of monolayer graphene, Corey et al. prepared graphene/polyvinylidene difluoride thin films and studied their electrothermal properties. The temperature coefficient of resistance changed slightly with the mass fraction, indicating that the electron transport mechanism of various mass fractions was the same [20]. Sun and others observed that there is a considerable drape effect in electronic transmission in graphene films, which increases the thickness of the graphene oxide thin film, compensating the unstable factors of graphene electronic transmission, and making the resistance–temperature characteristic of the graphene film sensor smoother; the temperature coefficient of resistance (TCR) was small but the stability of the sensor for very small temperature changes could lead to accurate detection [21]. Cai et al. fabricated an electrode based on microelectromechanical system (MEMS) technology, where oxidized graphene was inserted into the electrode fingers, detecting the change in the temperature of the capacitor [22]. De used inkjet printing technologies with single-layer graphene films, studied the resistance–temperature characteristic and response speed of the temperature of the mutations, observed that single-layer graphene showed a negative resistance temperature effect with a decrease in temperature resistance, and established a corresponding model, with the response speed of only 0.5 s, recovery rate of 10 s, and an ultra-high temperature response speed [23]. These studies show that temperature has a great influence on graphene resistance. However, the temperature effect is less considered in existing pressure sensor studies. In the application of graphene pressure sensors, temperature is a non-negligible factor. It is necessary to study the temperature characteristics of a pressure sensor based on BN/Graphene/BN heterostructure.

## 2. Theoretical Analysis

Electrophonon coupling is an important factor affecting the temperature sensitivity of graphene, and it is crucial to understand the metal–semiconductor properties of graphene [24]. The increase or decrease in graphene conductivity is closely related to the role of phonons [25]. From a microscopic perspective, the thermal stability of graphene depends on the strength of the C–C chemical bond. The carbon six-membered ring structure with strong bond energy in graphene can retain its stable structure at high temperatures. Theoretical and experimental results show that the thermal conductivity of graphene is up to 5300 W/m K [26], higher than that of carbon nanotubes (CNTs) and some other nano-level materials, indicating that graphene is suitable for high-performance thermal device materials [27]. Owing to its large specific surface area, graphene has a much larger area of contact heat than other carbon materials [28]. When graphene is heated, the surface folds will increase, and the electrophonon coupling rate will also change, leading to changes in the electrical properties of graphene; furthermore, its ultra-high thermal conductivity makes its response speed very fast.

When lattice vibration is considered, the atom (ion) deviates from the equilibrium position and causes a change in potential energy. The band electrons will be affected by the additional potential field generated by the lattice displacement, which is the interaction between the electrons and the lattice vibration. Electron–phonon interaction refers to the interaction between electrons and lattice vibrations. Positively charged atoms in a solid form a lattice at their static equilibrium, and the periodic field of the lattice causes the energy spectrum of the electron to exist as an energy band. In the periodic field, electrons have definite energy and the term “quasi momentum” k, where k is the wave vector, k = h/2π, and h is the Planck constant. It shows that the band electrons behave like free electrons. The action of the periodic field can be attributed to the effective mass of the electron. As the atoms are implicated in each other, the vibrations of the atoms form various lattice waves, namely, simple harmonic modes (normal modes) with different frequencies, wave vectors, and polarizations. The energy quantum of each normal mode is a phonon. Hence, the interaction between an electron and phonon represents the interaction between an electron and lattice vibration. It can be concluded from the Kubo–Greenwood formula that the conductivity of graphene decreases with an increase in temperature, and the effect of temperature on graphene alone will reduce the resistance of graphene.

Previous studies have shown that the influence of temperature on the conductivity of graphene involves a node that divides the entire temperature range into two parts [29]. It has been shown that the specific conductance of graphene decreases with an increase in temperature in the range 0–180 K and increases with an increase in temperature in the range 180–800 K [30]. When the temperature is below the node, the specific conductance of graphene decreases with an increase in temperature; when the temperature is above the node, the conductivity of graphene increases with an increase in temperature. We designed the BN/graphene/BN pressure sensors with and without a microcavity. The substrate thermally expands and stretches the BN/graphene/BN heterojunction, causing the sensor resistance to increase. The temperature itself reduces the resistance of graphene. Under the influence of temperature, the thermal expansion of the BN/graphene/BN pressure sensor with a microcavity is large, and the increasing trend of resistance caused by thermal expansion is much larger than that caused by the temperature itself, which leads to an increase in the resistance of the BN/graphene/BN pressure sensor with a microcavity. The thermal expansion of the BN/graphene/BN pressure sensor without a microcavity is small, and the increasing trend of resistance caused by thermal expansion is much smaller than that caused by the temperature itself, which leads to a reduction in the resistance of the BN/graphene/BN pressure sensor without a microcavity.

## 3. Fabrication of the Sensor

Graphene and boron nitride are mainly prepared on a copper base via chemical vapor deposition (CVD); subsequently, they are transferred to the target substrate through a wet transfer method and heterogeneous combination is completed. The thickness of the copper base is approximately 20 µm, the thickness of boron nitride is 13 nm, and boron nitride coverage is more than 90%. Boron nitride is transferred to the target via wet transfer. First, its surface is coated with poly (methyl methacrylate) (PMMA) to protect it from pollution and destruction. It is subsequently soaked in a ferric chloride solution for etching the copper base with a target base PMMA/BN film and cleaned. Finally, the transfer is completed and PMMA is removed. The graphene transfer process is also similar. Elastic thin films were prepared on a silicon/silicon dioxide substrate via inductively coupled plasma etching, and subsequently, graphene thin films were assembled on BN thin films via a fixed-point transfer technique. Subsequently, a BN/graphene/BN heterojunction was assembled and synthesized. Finally, photolithography, sputtering, and other MEMS processes were used to prepare the interconnected electrode and package structure.

The sensing unit of each graphene temperature sensor consists of a single layer of CVD graphene sandwiched between two layers of CVD h-BN. Using the wet transfer technique supported by large-area PMMA, two-dimensional heterogeneous structures were prepared on a Si/SiO_2_ wafer, and the graphene upper and lower layers were coated with metal to form an electrode contact. For the graphene pressure sensor with a cavity, first, the plasma-enhanced CVD (PECVD) technique was used to deposit SiO_2_ of thickness 1–2 µm on the bare Si wafer to form the device insulation layer. AZ5214 photoresist was used as the mask, and SF_6_, CHF_3_, and O_2_ were used to etch the cavity structure (depth of 500–700 nm) on SiO_2_. The bottom electrode of the device was prepared by magnetron sputtering Ti (10 nm)/Pt (50 nm) to form the bottom electrode contact of the sensitive unit. After the transfer, photolithography, and etching of the underlying h-BN and graphene, the top electrode of Au (50 nm) was prepared via electron beam evaporation. Finally, the transfer and graphics of the top h-BN were completed. Thus, the cavity-type graphene temperature sensor was fabricated. For the non-cavity-type graphene pressure sensor, the process of cavity etching is not required. The other processes are the same as those used for the cavity-type graphene temperature sensor.

**Figure 1:**
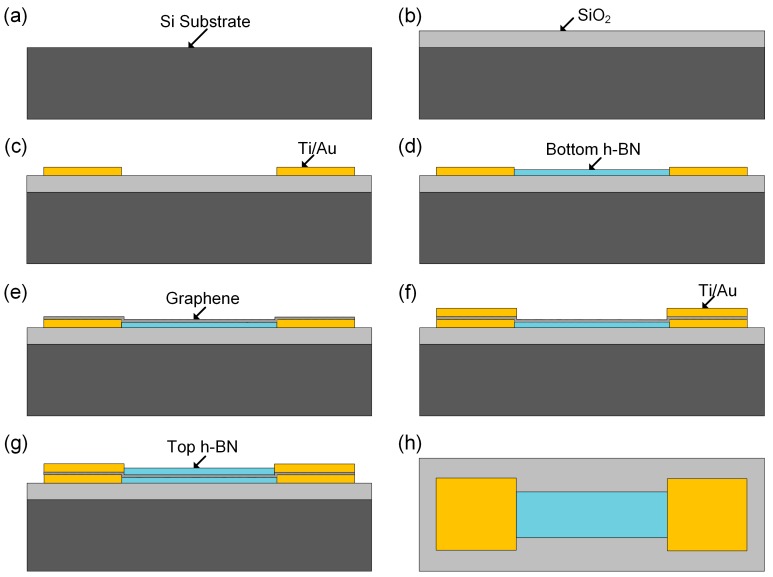
Structure and fabrication process of the sensors: the process flow of the non-cavity-type graphene sensor: (**a**). The silicon substrate is cleaned; (**b**). SiO_2_ insulating layer is formed on the surface of the silicon substrate through Plasma Enhanced Chemical Vapor Deposition (PECVD); (**c**). Sputtering of metal, forming a heterogeneous junction bottom electrode; (**d**). The underlying BN is transferred and the graphics of BN are completed; (**e**). Graphene is transferred and its graphics completed; (**f**). The top-level BN is transferred and the graphics of BN are completed; (**g**). The top metal electrode is vaporized; (**h**). Vertical view of the device structure.

**Figure 2:**
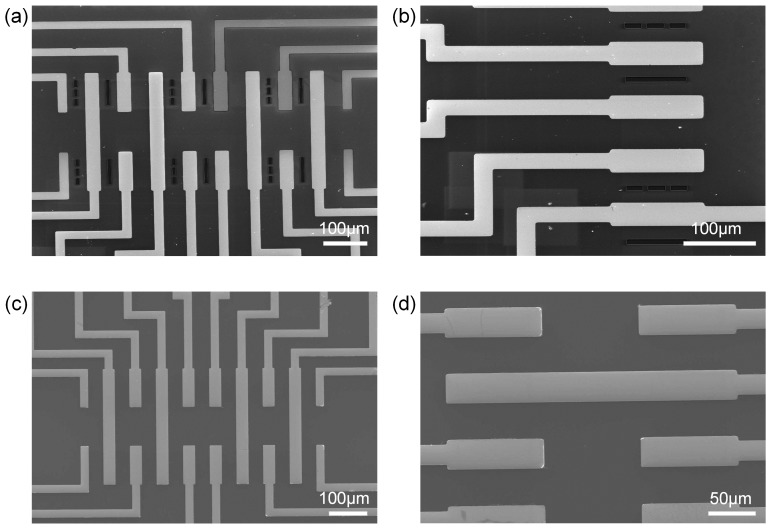
SEM images of the sensors: (**a**) Local diagram of the cavity-type graphene sensor chip; (**b**) Enlarged image of the bottom devices shown in (**a**); (**c**) Local diagram of a non-cavity-type graphene sensor chip; (**d**) Enlarged image of the bottom devices shown in (**c**).

The processing technology of the non-cavity-type graphene sensor is shown in Figure 1. The non-cavity-type sensor consists of Si as the substrate, SiO_2_ as the insulating layer, graphene wrapped by two layers of BN, and a Au electrode that forms ohmic contact with graphene. The processing technology of the cavity-type graphene sensor is described in [13]. In contrast to the non-cavity-type graphene sensor, a cavity of dimensions 64 × 6 µm is etched on SiO_2_. Scanning electron microscopy (SEM) diagrams of the two graphene sensors are shown in Figure 2.

**Figure 3:**
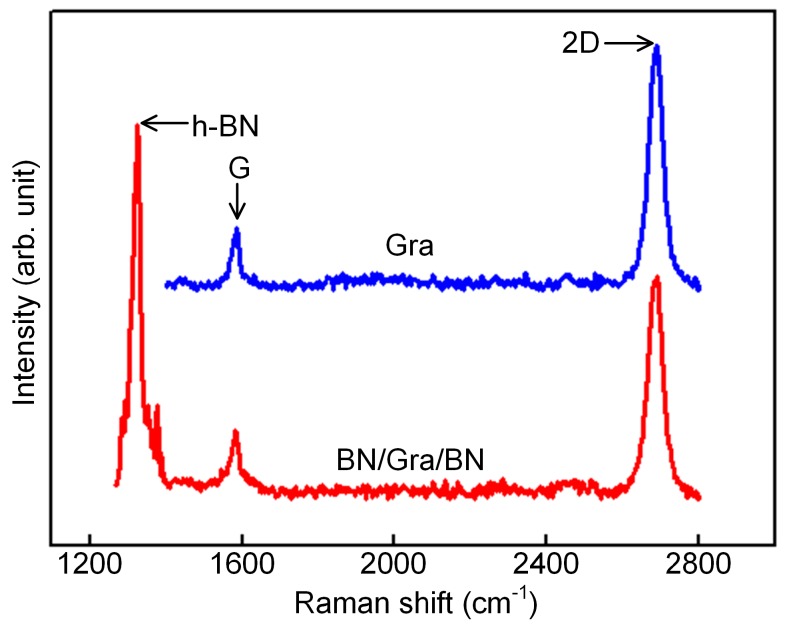
Raman test results of the BN/graphene/BN heterostructure: The intensity of peak G *I_G_* of graphene is 270, the peak *I_2D_* of graphene is 1050, and the peak strength ratio *I_2D_*/*I_G_* ≈ 3.89. The intensity of peak G *I_G_* of BN/graphene/BN is 296, the peak *I_2D_* of BN/graphene/BN is 958, and the peak strength ratio *I_2D_*/*I_G_* ≈ 3.24.

In terms of the transfer mass of graphene, it is crucial to determine its number of layers and quantify its disorder. Laser microscopic Raman spectroscopy is a standard and ideal analytical tool to characterize the above two properties. By obtaining the Raman spectrum of graphene, we can evaluate the structure and properties of graphene, such as the number of layers, stacking mode, defect number, edge structure, tension, and doping state. In addition, Raman spectroscopy plays an important role in clarifying the electronic phonon behavior of graphene. The Raman spectrum of graphene is composed of several peaks, mainly the G, D, and G’ peaks. The G peak is the main characteristic peak of graphene, which is caused by in-plane vibration of the carbon atom sp^2^, and it appears near 1580 cm^−1^. This peak can effectively reflect the number of layers of graphene, but it is easily affected by stress. The D peak is generally considered to be the disorderly vibration peak of graphene. The specific location of the peak is related to the laser wavelength. It is caused by the lattice vibration leaving the center of the Brillouin region and is used to characterize the structural defects or edges in graphene samples. The G’ peak, also known as the 2D peak, is the second-order Raman peak of double phonon resonance, which is used to characterize the interlayer stacking mode of carbon atoms in graphene samples. Its peak frequency is also affected by the laser wavelength. Specifically, monolayer graphene has two typical Raman characteristic peaks: the G peak near 1582 cm^−1^ and the 2D peak near 2700 cm^−1^. Furthermore, for graphene samples containing defects and with a certain degree of disorder, the D peak at approximately 1350 cm^−1^ is observed. The Raman spectrum characteristics of graphene materials can be determined by combining the appearance, peak strength, peak shape, peak position, and the relationship between different Raman characteristic peaks. Raman characterization showed that BN protection had no effect on the properties of graphene. We performed Raman test on the monolayer graphene and the BN/graphene/BN heterostructure, as shown in Figure 3. The results show that boron nitride has little effect on graphene performance, and graphene performance is still excellent. 

**Figure 4:**
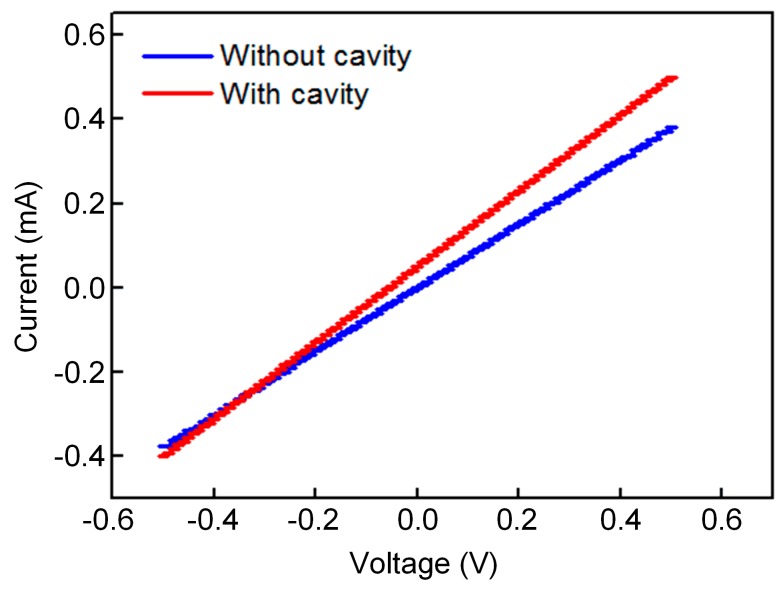
Electrical characterization of the BN/graphene/BN pressure sensor: The resistance of the BN/graphene/BN pressure sensor with a microcavity is approximately 1092 ± 4.6 Ωsq^−1^, and that of the BN/graphene/BN pressure sensor without a microcavity is approximately 1320 ± 6.5 Ωsq^−1^.

We measured the current (*I*)–voltage (*V*) characteristic using a four-probe tester. Six samples of each structure were measured. The resistances of the BN/graphene/BN pressure sensor with a microcavity are approximately 1050 Ωsq^−1^, 1092 Ωsq^−1^, 1065 Ωsq^−1^, 1105 Ωsq^−1^, 1083 Ωsq^−1^, 1170 Ωsq^−1^ and that of the BN/graphene/BN pressure sensor without a microcavity are approximately 1311 Ωsq^−1^, 1320 Ωsq^−1^, 1330 Ωsq^−1^, 1350 Ωsq^−1^, 1380 Ωsq^−1^, 1400 Ωsq^−1^. The *I–V* curve of the BN/graphene/BN pressure sensors indicates that graphene retains its high quality after the top BN protective layer is coated. The electrical characterization of one of the selected samples is shown in Figure 4.

## 4. Temperature Characteristic Measurement

**Figure 5:**
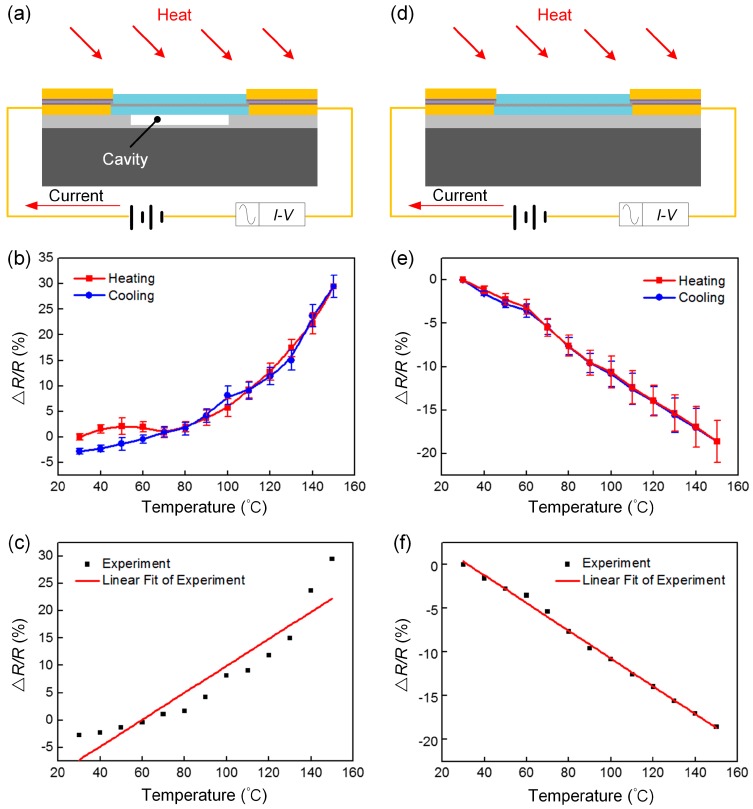
Experimental characterizations of the BN/graphene/BN pressure sensors: (**a**) test structure diagram of the pressure sensor with a cavity; (**b**) the fractional change in the electrical resistivity variation of the BN/graphene/BN pressure sensor with a microcavity over the range 30–150 °C; (**c**) the fitting curve of the BN/graphene/BN pressure sensor with a microcavity over the range 30–150 °C; (**d**) test structure diagram of the pressure sensor without a cavity; (**e**) the fractional change in the electrical resistivity variation of the BN/graphene/BN pressure sensor without a microcavity over the range 30–150 °C; (**f**) the fitting curve of the BN/graphene/BN pressure sensor without a microcavity over the range 30–150 °C.

To test the temperature characteristic of the two pressure sensors, the resistance change was measured using a high-temperature drying oven with a change in temperature from 30 to 150 °C. Six samples of each structure were measured, and every sample underwent three heating and cooling experiments. The resistance change was recorded with each measurement in steps of 10 °C, and the temperature was maintained at each step of 10 °C for 25 min to observe the change in resistance. The test structure of the BN/graphene/BN pressure sensor with a microcavity is shown in Figure 5a, and the test structure of the BN/graphene/BN pressure sensor without a microcavity is shown in Figure 5d. Figure 5b shows the fractional change in the electrical resistivity variation of the BN/graphene/BN pressure sensor with a microcavity over the range 30–150 °C. Figure 5c shows the fitting curve of the BN/graphene/BN pressure sensor with a microcavity over the range 30–150 °C. Figure 5e shows the fractional change in the electrical resistivity variation of the BN/graphene/BN pressure sensor without a microcavity over the range 30–150 °C. Figure 5f shows the fitting curve of the BN/graphene/BN pressure sensor without a microcavity over the range 30–150 °C. The TCR is often used to describe the temperature-sensitive properties and is popularly known as sensitivity [31]. Where *R*_0_, *R*, and △*T* are the resistance at the initial temperature T_0_, the resistance at temperature T, and the D-value between the temperature T and the initial temperature T_0_.

(1) $$\mathrm{TCR}=\left(R-R_{0}\right)/\left(R_{0}\cdot \Delta T\right),$$

As a temperature-sensitive material, BN/graphene/BN has a higher temperature coefficient, and can be used as a graphene pressure sensor temperature compensation module. The BN/graphene/BN pressure sensor with a microcavity has a positive temperature coefficient, high temperature sensitivity, and poor linearity, with a linear standard error of 2.66046. The BN/graphene/BN pressure sensor without a microcavity has a negative temperature coefficient, and low temperature sensitivity relative to the cavity, but it has high linearity, with a linear standard error of 0.27858. A comprehensive comparison of the BN/graphene/BN pressure sensors shows that the sensor without a microcavity has better temperature characteristics, apart from the graphene pressure sensor for temperature compensation. The visualized parameters of the BN/Gra/BN pressure sensors with and without a cavity that we designed as well as other temperature sensors are shown in Table 1. From this table, we conclude that the BN/Gra/BN pressure sensors with and without a cavity that we made has a higher sensitivity compared to other sensors and it can be tested at temperatures up to 150 °C.

## 5. Conclusions

For two different structures of BN/graphene/BN sensors, this article presents an analysis of the thermal expansion and the influence of electrophonon coupling on the temperature properties of graphene. When the effect of thermal expansion is greater than that of electrophonon coupling, the resistance of the BN/graphene/BN pressure sensor increases with an increase in temperature. The results show that, for the BN/graphene/BN pressure sensor with a microcavity, the thermal expansion effect is greater than the influence of electrophonon coupling, and the sensor temperature coefficient is positive with a value of 0.25%°C^−1^, whereas for the BN/graphene/BN pressure sensor without a microcavity, the thermal expansion effect is smaller than the influence of electrophonon coupling, and the sensor temperature coefficient is negative, with a value of −0.16%°C^−1^. The two different structures present contrasting conclusions. The BN/graphene/BN pressure sensor with a microcavity shows good temperature sensitivity and the BN/graphene/BN pressure sensor without a microcavity shows good linearity. These results indicate that the influence of temperature on the pressure sensors based on BN/graphene/BN heterostructures should be considered, especially for devices with pressure microcavities. BN/graphene/BN heterostructures without microcavities can be used as high-performance temperature sensors, facilitating the design of a high-temperature graphene pressure sensor.

## Figures and Tables

**Table 1:** Some types of temperature sensors and their performances.

Sensor Type	Temperature Coefficient	References
The BN/Gra/BN pressure sensors with a cavity	0.25%°C^−1^	This work
The BN/Gra/BN pressure sensors without a cavity	−0.16%°C^−1^	This work
Monolayer graphene	0.10%°C^−1^	[32]
Multi-walled CNTs	−0.13%°C^−1^	[33]
Graphite	0.05%°C^−1^	[34]
CNTs prepared by CVD	0.04%°C^−1^	[35]
